# Pathology of carbon monoxide poisoning in two cats

**DOI:** 10.1186/s12917-018-1385-4

**Published:** 2018-03-05

**Authors:** Arya Sobhakumari, Robert H. Poppenga, J. Brad Pesavento, Francisco A. Uzal

**Affiliations:** 1 California Animal Health and Food Safety Laboratory System, Davis branch, School of Veterinary Medicine, University of California Davis, Davis, USA; 2 California Animal Health and Food Safety Laboratory System, San Bernardino branch, School of Veterinary Medicine, University of California Davis, 105 W Central Ave, San Bernardino, CA 92408 USA

**Keywords:** Carbon monoxide, Cats, COHb %, Forensic investigation

## Abstract

**Background:**

Carbon monoxide (CO), a common cause of poisoning in human beings has also been implicated in the death of animals. Though there are multiple studies on CO poisoning and relevant lethal blood COHb concentrations in humans, there are no reliable reports of diagnostic lethal carboxyhemoglobin percentage of saturation (COHb%) in cats. Additionally, due to shared housing environments, exposures to companion animals can be a surrogate for lethal exposures in human beings and provide valuable information in concurrent forensic investigations.

**Case presentation:**

Two adult Singapura brown ticked cats were submitted to the California Animal Health and Food Safety Laboratory (CAHFS) for necropsy and diagnostic work-up. These animals were found dead along with their two deceased owners. Similar lesions were observed in both cats. At necropsy, gross lesions consisted of multifocal, large, irregular, bright red spots on the skin of the abdomen and the inner surface of ear pinnae, bright red muscles and blood. The carcasses, and tissues fixed in formalin retained the bright red discoloration for up to two weeks. Microscopic lesions included diffuse pulmonary congestion and edema, and multifocal intense basophilia of cardiomyocytes mostly affecting whole fibers or occasionally a portion of the fiber. Based on the clinical history,gross and microscopic changes, cyanide or carbon monoxide poisoning was suspected. Blood samples analyzed for carbon monoxide showed 57 and 41% carboxyhemoglobin COHb%. Muscle samples were negative for cyanide.

**Conclusion:**

There are no established reference values for lethal COHb concentration in cats. The COHb % values detected in this case which fell within the lethal range reported for other species, along with the gross lesions and unique histological findings in the heart suggest a helpful criteria for diagnosis of CO intoxication associated death in cats. This case demonstrates that since pets share the same environment as human beings and often are a part of their activities, they can be useful adjuncts in potential forensic investigations to help solve human cases.

## Background

Carbon monoxide (CO) poisoning has been frequently described in human beings. However, information about CO poisoning and associated lesions in animals is scant to non-existent, especially in cats. In particular, there are no reports of diagnostic lethal carboxyhemoglobin percentage of saturation (COHb%) in cats and the threshold for toxicity in this species has not been established. It is possible that animal cases remain undiagnosed due to lack of adequate testing opportunities in veterinary diagnostic laboratories. The main reason for CO poisoning in human beings is incomplete combustion of hydrocarbons in non-ventilated buildings, associated with faulty furnaces and house fires. This seems to be the same mechanism for CO poisoning in companion animals such as dogs and cats, because of their shared environment with humans.

CO has more than 210 times greater affinity for hemoglobin than oxygen (O_2_) [1], readily forming carboxyhemoglobin (COHb), thus reducing the oxygen carrying capacity of red blood cells and leading to hypoxemia and tissue hypoxia. Formation of COHb not only displaces oxygen but CO causes a conformational change in hemoglobin after the binding, resulting in greater affinity for oxygen in the remaining heme moieties. This leads to a shift to left of the O_2_ dissociation curve and reduced delivery of O_2_ to tissues [2, 3]. CO has high affinity for myoglobin and binding to cardiac myoglobin can cause myocardial depression, hypotension and arrhythmias. Cardiac decompensation results in further tissue hypoxia which is the ultimate cause of death [4].

Current scientific literature on spontaneous CO intoxication in small animals is limited. While a few papers have been published describing spontaneous CO intoxication in dogs [3, 5, 6], there are no reports that covered gross and histological lesions in various organs in cats. There is only one report on respiratory distress upon CO poisoning in cats, although pathology of the intoxication was not described in that report [7].

This case describes for the first time in detail the gross, microscopic and toxicological findings, specifically the lethal blood COHb concentration, in two cats that died following CO intoxication. This report also indicates the utility of postmortem blood as a feasible sample for diagnostic confirmation. Additionally, it emphasizes the significance of testing animals as a useful adjunct to concurrent human forensic investigations since pets share the same environment as owners and face similar risks with respect to exposure to various chemicals, including CO.

## Case presentation

Two adult Singapura brown ticked cats (a spayed female and a neutered male) were submitted to the San Bernardino branch of CAHFS for necropsy and diagnostic work up. The cats had been found dead in a household together with their two deceased owners. The exact date of death was unknown.

**Fig. 1:**
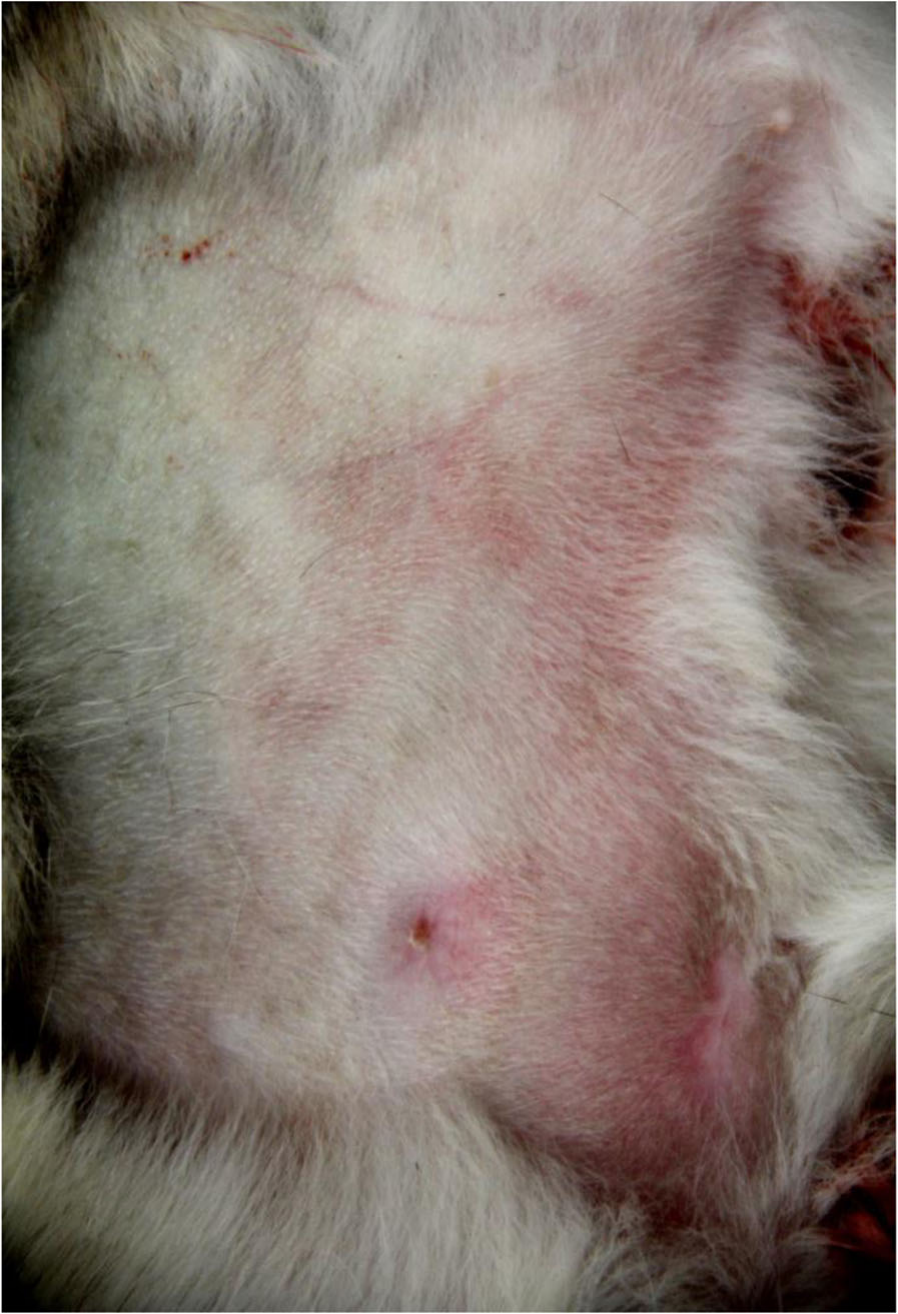
Gross findings in a cat with carbon monoxide intoxication. Bright red discoloration on the skin of the abdomen

**Fig. 2:**
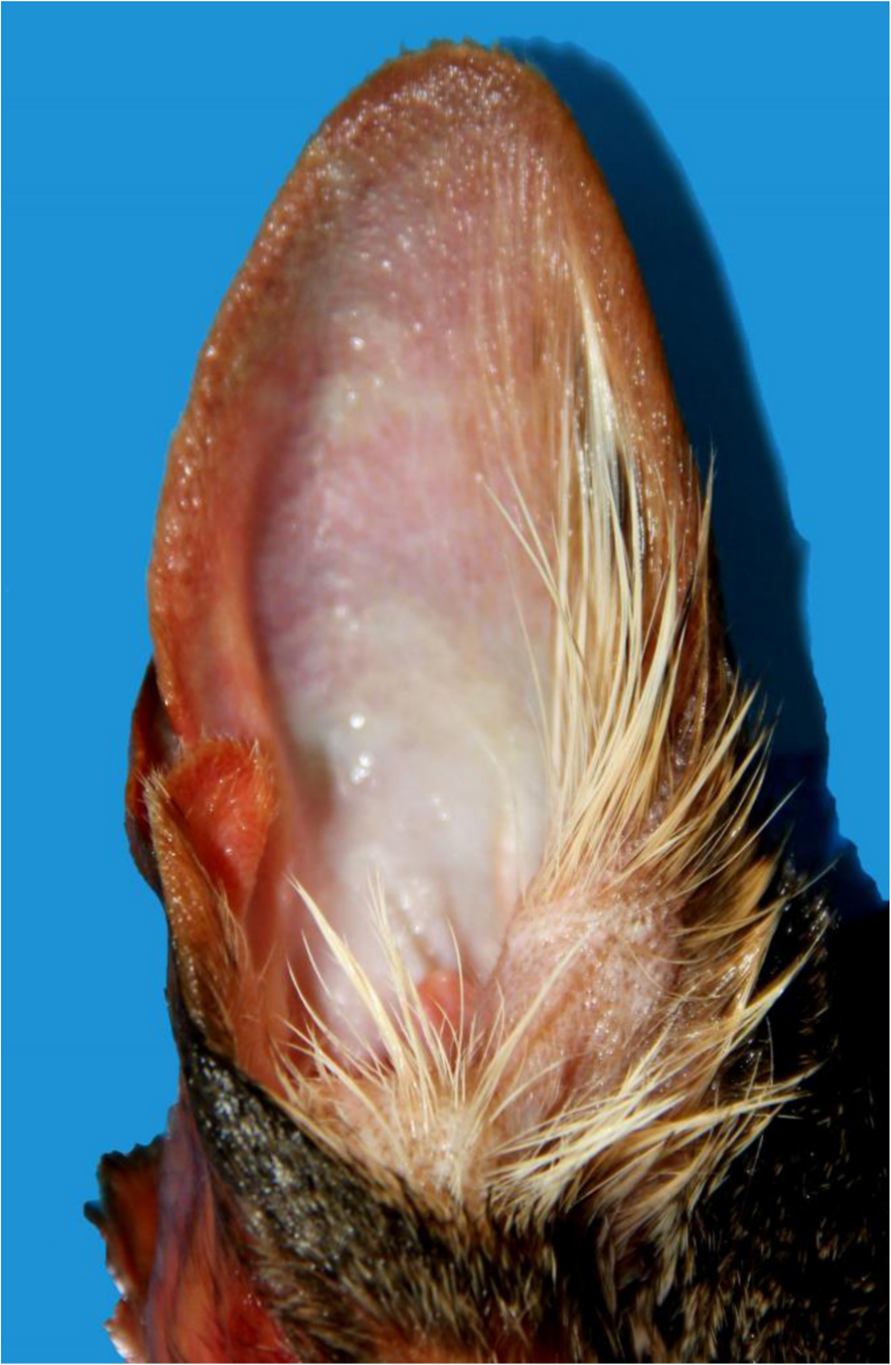
Gross findings in a cat with carbon monoxide intoxication. Bright red discoloration on the inner surface of the ear pinna

Grossly, both cat carcasses were in good nutritional condition and moderate state of postmortem decomposition. The stomach of cat A was almost empty except for ~ 2 mls of mucous fluid and the stomach of cat B had a ~ 50 mls of semi digested kibble. Both cats presented the following gross abnormalities: bright pink-red discoloration of the aqueous fluid (this change was most marked in Cat A), multifocally bright red discoloration on the skin of the abdomen (Fig. 1) and inner surface of the ear pinnae (Fig. 2), bright red skeletal muscles, congestion and bright red discoloration of the abdominal serosa, congested and edematous lungs, mild hydrothorax and hydropericardium. No other significant gross abnormalities were observed in either carcasses. In particular, no gross abnormalities were observed in the brain of either cat.

**Fig. 3:**
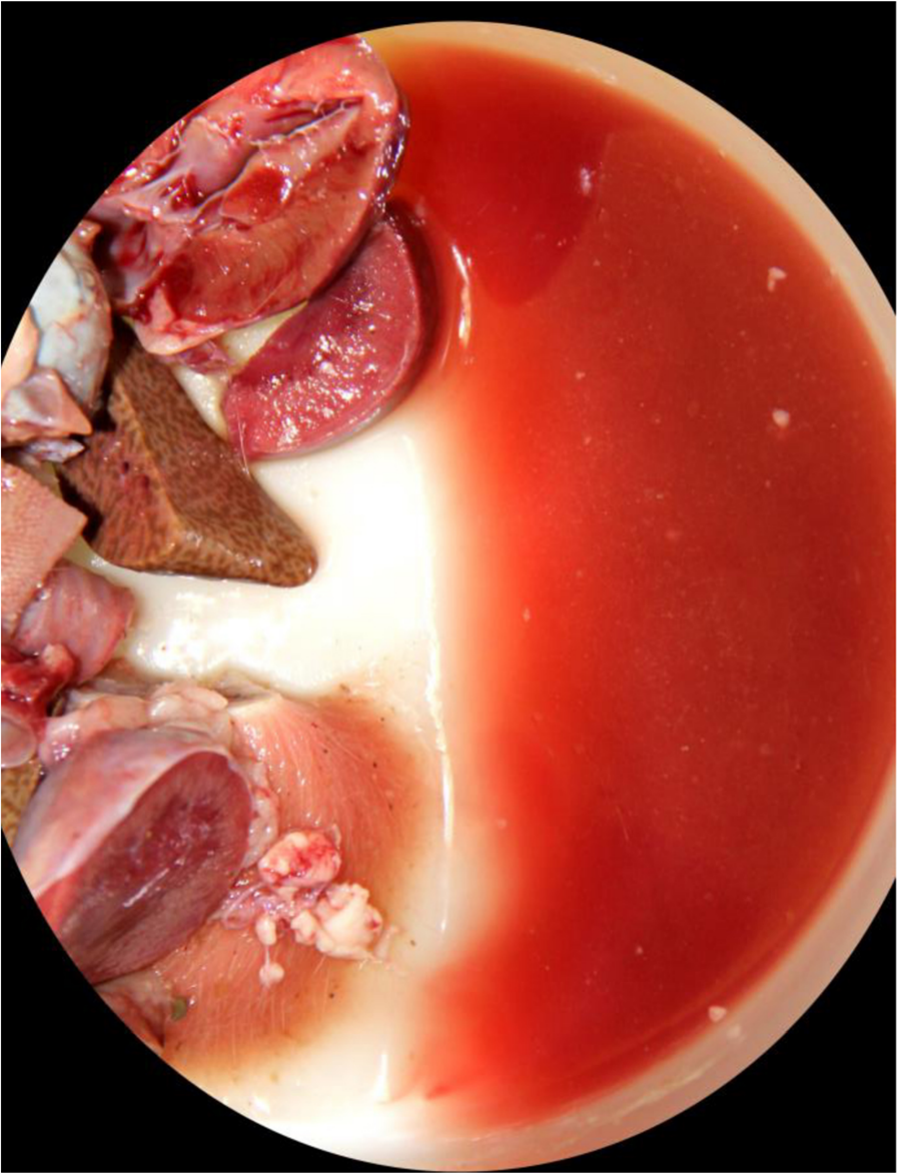
Gross findings in a cat with carbon monoxide intoxication. Bright red discolored formalin where tissues were immersed for 2 weeks

After necropsy, the carcasses were stored refrigerated for two weeks before they were submitted for cremation, and they retained the bright red discoloration for the whole period (Fig. 3). Formalin fixed tissues and the formalin in which tissues were immersed, retained similar discoloration for the first two weeks after collection; this discoloration faded progressively after that.

Tissue samples from most organs of both cats, including representative areas of the brain (cortex, corpus striatum, thalamus, midbrain, cerebellum and medulla), were routinely processed for the production of HE stained sections. Selected sections of heart from both animals were also stained with Von Kossa to investigate possible calcium deposits and Phosphotungstic acid hematoxylin (PTHA) to highlight the structure of myocardiocytes.

**Fig. 4:**
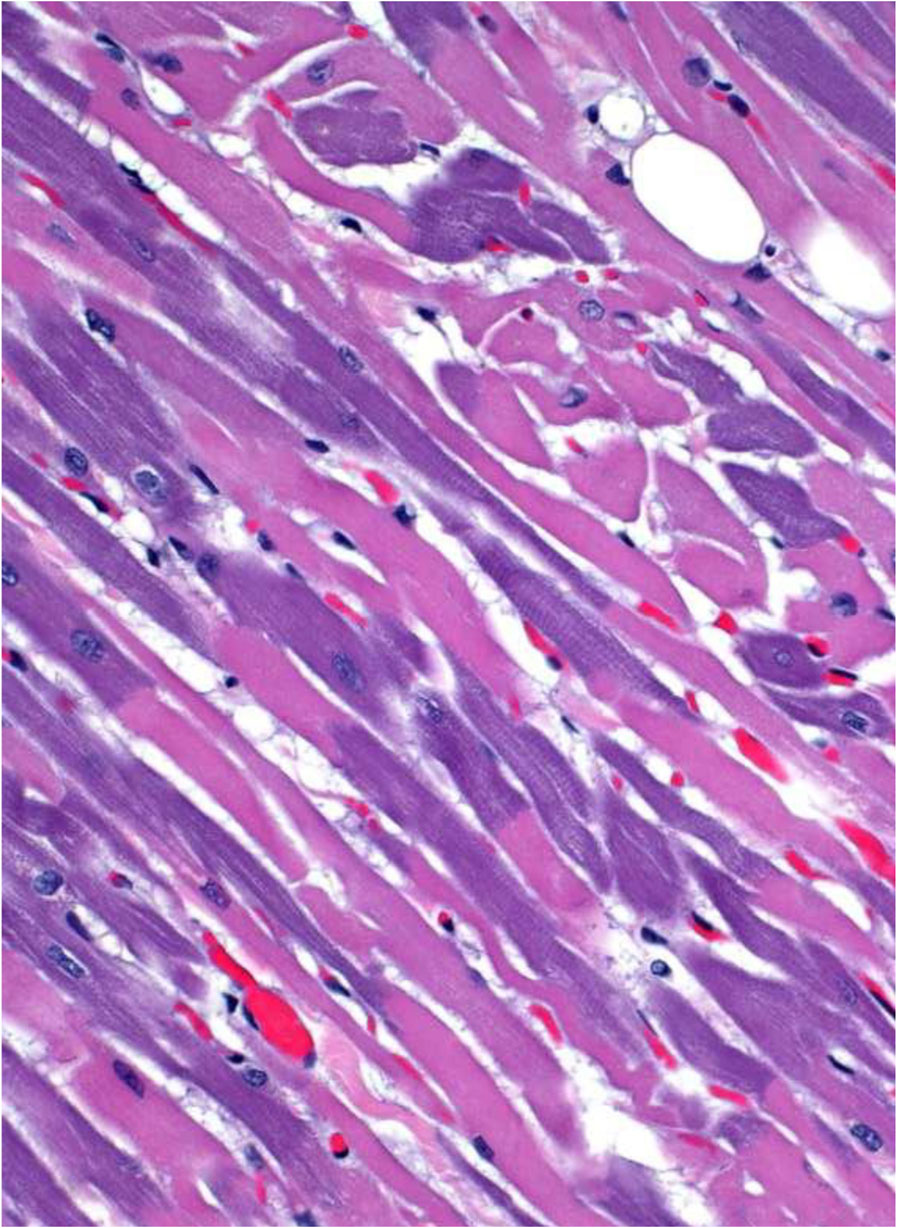
Microscopic findings in a cat with carbon monoxide intoxication. Multifocal basophilia in cardyomyocytes. HE, 250X

Microscopic lesions were similar in both cats and included diffuse pulmonary congestion, interstitial and alveolar edema, and multifocal intense basophilia of cardiomyocytes (Fig. 4). The latter mostly affected whole fibers but occasionally only a portion of the fiber, with a clear transverse line of demarcation from the rest of the fiber. Rarely, discrete areas of hypercontraction bands were seen in individual cardiomyocytes; these lesions were visible on HE sections but were highlighted on PTHH stained sections. Examination of Von Kossa stained sections of the heart was unremarkable. No other significant microscopic abnormalities were observed in any of these cats. Transmission electron microscopy of formalin fixed heart from both cats was performed but was unrewarding.

Based on the clinical history, gross and microscopic changes, cyanide or carbon monoxide poisoning was suspected. Skeletal muscle from both cats was analyzed for cyanide by a distillation method as previously described [8]. Briefly, cyanide in the tissues was converted to hydrogen cyanide gas which was chlorinated and converted to a colored dye. The absorbance was then measured spectrophotometrically at 578 nm and cyanide quantitated from a standard curve. Cyanide was not detected in the muscle of either cat at or above a reporting limit of 1 ppm.

Since the cyanide test was negative, postmortem blood samples were analyzed for CO by a modification of the Comopac® electrochemical gas meter as previously described [9]. Briefly, blood was mixed with saturated sodium chloride in a screw top glass vial adapted to fit onto a Genesis® portable CO monitor (Thermo Scientific) and CO, liberated in a closed headspace, was measured for 5 min. The Hb was measured by an azidemethemoglobin method using HemoCue® Hb 201 System. The blood COHb was reported as % saturation of Hb with CO. COHb saturation was 57% and 41% for cats A and B, respectively.

## Discussion and conclusions

In this case, a presumptive diagnosis of CO intoxication was established based on the clinical history, gross and microscopic findings. Gross and microscopic changes have not been described before in cats intoxicated with carbon dioxide. In humans and other animals intoxicated with CO bright red discoloration of skin, mucosas and muscle, cherry red color of the blood and brain changes related to anoxia, including necrosis in the cortex and white matter of cerebral hemispheres, globus pallidus and brainstem have been described and are suggestive, but non-specific for this intoxication [3, 5, 6]. These changes are not, however, considered pathognomonic and confirmation of the diagnosis should be based on detection of toxic levels of COHb in the blood of cadavers, which is standard practice in human forensic medicine.

In this case, both cats had gross findings considered compatible with carbon monoxide intoxication in humans and dogs [3, 5, 6]. In addition, bot animals had blood values of COHb which are considered toxic for humans and, in absence of reference values for cats, this finding was used to confirm the diagnosis of CO intoxication.

Brain changes have been described in human beings and dogs intoxicated with carbon monoxide before. In the cats presented here, no gross or microscopic lesions were observed in the brain. This might be related to a very acute clinical course with no time for central nervous system lesion development. This is, however, only speculation, as clinical information was not available on these cats which were found dead.

The microscopic discoloration of cardiomyocytes of these two cats is an unusual lesion that has not been described before in humans or animals intoxicated with CO. Because no ultrastructural lesions were observed in the heart of either cat, the pathogenesis of this discoloration could not be determined; it is possible that biochemical alterations not accompanied by morphological (microscopic or ultrastructural) changes occurred associated with CO intoxication.

CO is a colorless, odorless, non-irritant gas produced by incomplete combustion of fuels and is a leading cause of human poisoning and mortality in the US [10]. Two mechanisms of action (acute and delayed) exist for CO intoxication. The formation of COHb in intoxicated individuals decreases the oxygen carrying capacity of hemoglobin and shifts the O_2_ dissociation curve to the left impairing O_2_ release resulting in tissue hypoxia, which is mainly responsible for the clinical signs of CO poisoning. However, it has been suggested that the above mechanism alone does not account for the toxicity of CO since in many instances the concentration of COHb does not correlate with the clinical signs. In an early study, when dogs were exposed to 13% CO in atmospheric air, the animals died within one hour and had COHb concentrations of 54-90% [5]. However, the transfusion of 80% COHb saturated blood to healthy recipient dogs which resulted in a similar 57 – 64% COHb in their blood did not cause intoxication [5, 11]. It is suggested that acute toxicity of CO may be the result of binding of CO to heme proteins other than hemoglobin such as cytochromes and myoglobin, which interferes with cellular respiration and generation of free radicals through disruption of oxidative metabolism [12, 13]. Additionally, CO has been found to stimulate guanylyl cyclase resulting in relaxation of vascular smooth muscles, cerebral vasodilation and loss of consciousness [6, 14]. CO also displaces nitric oxide (NO) from platelets leading to peroxynitrite formation, endothelial damage, leukocyte adhesion, formation of free radicals, and lipid peroxidation in the brain microvasculature which may be a mechanism for delayed neurological sequelae [15].

Levels of COHb have been measured in many species both at baseline and associated with lethality. In human beings, clinical signs of CO toxicity start at 20% COHb and death occurs between 50 and 80% COHb [16]. In dogs mortality was reported to occur within one hour when exposed to 13% CO in atmospheric air with subsequent COHb concentrations of 54-90% [5]. Ashbaugh measured COHb in healthy, clinically normal dogs and fire victim dogs, and found that the values ranged between 5.6 - 6.4% and 8.3 - 37%, respectively [17]. COHb concentration has been reported in two cats that became ataxic and tachypneic following exposure to generator fumes in a closed warehouse for 8 h [7]. These animals had COHb concentrations of 5 and 9% which declined to baselines levels of 0-4% after oxygen therapy and supportive treatment. However, there are no guidelines to interpret lethal COHb concentrations in blood of cats intoxicated with CO. In the present case, the concentration of COHb in both the cats fell within the lethal ranges reported for dogs and humans. Since no previous data was available to compare, we concluded that the detected concentrations are lethal for cats in the context of the characteristic lesions.

Although measurement of blood COHb is used as a diagnostic tool for CO poisoning, it may not fully correlate with the severity of the symptoms. The COHb in blood is not an absolute index of compromised oxygen delivery at the tissue level. Additionally, unmeasured CO uptake in tissues, which is hypothesized to increase during hypoxia because of the competition of CO and O2 on the binding site of hemoproteins also contributes to the clinical signs. Therefore, the limitation of COHb levels is that it can be used to guide therapeutic strategies but not predict treatment outcomes [7, 18].

The incidence of CO intoxication in pets is unknown. The Pet Poison Helpline (St. Paul, Minnesota) receives an average of 3 – 4 suspected or confirmed cases of CO poisoning every year, with a potential for many suspicious cases unconfirmed due to lack of adequate diagnostic facilities.

Because of the proximity of pets to human beings, investigation of morbidity or mortality can be valuable in concurrent human forensic investigations. The use of dog and cat hair DNA, tissues, saliva and pet food remnants have become more common in criminal investigations helping solve numerous cases [19, 20]. An unusual case of apparent suicide/homicide involving two human beings and a dog was described where the cause of death was identified to be diazepam overdose which was later detected in the liver tissue and dog food remnants [21]. In the present case, the presumed sudden death of both human beings and the cats in the same environment bolsters the argument that a multidisciplinary approach involving pets would be more likely to yield successful investigational outcomes since both species were likely exposed to the same toxicant.

## Abbreviations

CAHFS: California Animal Health and Food Safety Laboratory
CO: Carbon monoxide
COHb: Carboxyhemoglobin
COHb%: Carboxyhemoglobin percentage of saturation
HB: Hemoglobin
mls: Milliliters
O_2_: Oxygen
ppm: Parts per million
PTAH: Phosphotungstic acid hematoxylin
